# Mobile App for Improved Self-Management of Type 2 Diabetes: Multicenter Pragmatic Randomized Controlled Trial

**DOI:** 10.2196/10321

**Published:** 2019-01-10

**Authors:** Payal Agarwal, Geetha Mukerji, Laura Desveaux, Noah M Ivers, Onil Bhattacharyya, Jennifer M Hensel, James Shaw, Zachary Bouck, Trevor Jamieson, Nike Onabajo, Madeline Cooper, Husayn Marani, Lianne Jeffs, R Sacha Bhatia

**Affiliations:** 1 Women’s College Hospital Institute for Health System Solutions and Virtual Care Women’s College Hospital Toronto, ON Canada; 2 Department of Family and Community Medicine University of Toronto Toronto, ON Canada; 3 Department of Medicine University of Toronto Toronto, ON Canada; 4 Institute for Health Policy, Management and Evaluation University of Toronto Toronto, ON Canada; 5 Women’s College Research Institute Women’s College Hospital Toronto, ON Canada; 6 Department of Psychiatry University of Manitoba Winnipeg, MB Canada; 7 Department of Psychiatry University of Toronto Toronto, ON Canada; 8 Division of General Internal Medicine St Michael’s Hospital Toronto, ON Canada; 9 Li Ka Shing Knowledge Institute St. Michael’s Hospital Toronto, ON Canada

**Keywords:** mobile apps, diabetes mellitus, type 2, self-management, blood glucose self-monitoring, randomized controlled trial, pragmatic clinical trial

## Abstract

**Background:**

As the increasing prevalence of type 2 diabetes mellitus has put pressure on health systems to appropriately manage these patients, there have been a growing number of mobile apps designed to improve the self-management of diabetes. One such app, BlueStar, has been shown to significantly reduce hemoglobin A_1c_ (HbA_1c_) levels in small studies and is the first app in the United States to receive Food and Drug Administration approval as a mobile prescription therapy. However, the impact of the app across *real-world* population among different clinical sites and health systems remains unclear.

**Objective:**

The primary objective of this study was to conduct a pragmatic randomized controlled trial of the BlueStar mobile app to determine if app usage leads to improved HbA_1c_ levels among diverse participants in real-life clinical contexts. We hypothesized that this mobile app would improve self-management and HbA_1c_ levels compared with controls.

**Methods:**

The study consisted of a multicenter pragmatic randomized controlled trial. Overall, 110 participants randomized to the immediate treatment group (ITG) received the intervention for 6 months, and 113 participants randomized to the wait-list control (WLC) group received usual care for the first 3 months and then received the intervention for 3 months. The primary outcome was glucose control measured by HbA_1c_ levels at 3 months. Secondary outcomes assessed intervention impact on patient self-management, experience of care, and self-reported health utilization using validated scales, including the Problem Areas in Diabetes, the Summary of Diabetes Self-Care Activities, and the EuroQol-5D. Intervention usage data were collected directly from the app.

**Results:**

The results of an analysis of covariance controlling for baseline HbA_1c_ levels did not show evidence of intervention impact on HbA_1c_ levels at 3 months (mean difference [ITG−WLC] −0.42, 95% CI −1.05 to 0.21; *P*=.19). Similarly, there was no intervention effect on secondary outcomes measuring diabetes self-efficacy, quality of life, and health care utilization behaviors. An exploratory analysis of 57 ITG participants investigating the impact of app usage on HbA_1c_ levels showed that each additional day of app use corresponded with a 0.016-point decrease in participants’ 3-month HbA_1c_ levels (95% CI −0.03 to −0.003). App usage varied significantly by site, as participants from 1 site logged in to the app a median of 36 days over 14 weeks (interquartile range [IQR] 10.5-124); those at another site used the app significantly less (median 9; IQR 6-51).

**Conclusions:**

The results showed no difference between intervention and control arms for the primary clinical outcome of glycemic control measured by HbA_1c_ levels. Although there was low usage of the app among participants, results indicate contextual factors, particularly site, had a significant impact on overall usage. Future research into the patient and site-specific factors that increase app utilization are needed.

**Trial Registration:**

Clinicaltrials.gov NCT02813343; https://clinicaltrials.gov/ct2/show/NCT02813343 (Archived by WebCite at https://clinicaltrials.gov/ct2/show/NCT02813343)

## Introduction

The worldwide burden of type 2 diabetes mellitus (T2DM) continues to increase, with almost 9% of the global population expected to have T2DM by 2035 [1]. The increasing prevalence of T2DM will put pressure on health systems to appropriately manage these patients to avoid diabetic complications. Optimizing self-management of glycemic control and other risk factors in conjunction with pharmacologic therapy may be an efficient way to improve patient outcomes [2-5]. Although self-management is traditionally offered through in-person educational programs, this is resource intensive, and advances in mobile technology provide the opportunity to deliver effective self-management support to patients that is convenient and potentially cost-effective [6-9].

There are a growing number of mobile apps designed to improve the self-management of T2DM patients [10-12], although few have been rigorously evaluated. One diabetes management app, called BlueStar, a smartphone-enabled app that is designed to serve as a virtual coach for patients, has been shown to significantly reduce hemoglobin A_1c_ (HbA_1c_) levels in T2DM patients, seen by primary care physicians [13]. As a result, BlueStar is the first app in the United States to be given Food and Drug Administration approval as a mobile prescription therapy [14]. Previous studies using BlueStar were small however and conducted on a relatively homogenous patient population [13]. As a result, it remains unknown whether the result of these studies would be generalizable to a diverse *real-world* population across different clinical sites. In addition, multiple studies of mobile apps for chronic diseases have highlighted the importance of contextual and implementation factors, including clinician training, integration into existing workflows, and ongoing clinician engagement with the patient as important influencers of clinical outcomes [15], yet previous studies were not designed to assess these factors.

The purpose of this study was to conduct a pragmatic randomized controlled trial of the BlueStar mobile app on T2DM patients with poorly controlled blood sugar to determine if the use of the app would lead to improved HbA_1c_ levels compared with controls in real-life clinical contexts. We hypothesized that this mobile app would improve patient self-management, and ultimately, patients with the app would have improved HbA_1c_ levels compared with controls.

## Methods

### Settings

Participants were recruited from 3 hospital-based diabetes education programs (DEPs) in Ontario, Canada. Most health services in Ontario, Canada, are financed through the publicly funded Ontario Health Insurance Program (OHIP), which covers medically necessary services delivered by physicians, including primary, specialty, and emergency care. Patients with T2DM typically get most of their diabetes care in short visits from family physicians who may or may not have additional multidisciplinary support. In addition, OHIP covers services provided by DEPs, which are multidisciplinary, nonphysician -led programs designed to deliver self-management education of diabetes and self-management support [16]. The 3 recruitment sites included (1) a DEP located in an urban area in a large city center (>2 million people), (2) 1 located in a midsize city in a remote area of the province (<150,000 people), and (3) 1 located in a semiurban area surrounding a large city center (<600,000 people). These sites serve a diverse range of patients including a large immigrant community, rural patients, and a large Aboriginal population. The services of these programs are complementary to primary care delivered through the patients’ primary care provider (PCP) and usually do not include medication titration.

### Trial Design

The study consisted of a multicenter, pragmatic randomized controlled trial with blinded outcome assessment designed to evaluate the effectiveness of the BlueStar app. A full description of the protocol has been previously published [17]. Participants with an HbA_1c_ level higher than 8.0% were recruited from the 3 DEPs, where they received support for diabetes management and randomized in a ratio of 1:1 to 2 groups: (1) immediate treatment group (ITG) or (2) wait-list control (WLC) group. The ITG received the intervention immediately for a total duration of 6 months. The WLC group received usual care for the first 3 months, at which point they received the intervention and used the app for a total of 3 months. Outcomes were measured at baseline as well as 3 and 6 months.

### Participants

Participants were eligible for inclusion in the study if they met the following criteria: (1) adults aged older than 18 years; (2) obtaining care for T2DM at a participating DEP; (3) HbA_1c_ ≥8.0% (and at least 1% above the participant’s target level) on most recent laboratory report within the last 3 months; (4) currently using an active email address or able and willing to obtain one; and (5) able to read the English language (self-reported). Patients were excluded if they have type 1 diabetes, were on continuous glucose monitoring, had an insulin pump, were on dialysis, pregnant, or are unable to use a computer or mobile phone because of severe mental or physical impairment.

### Recruitment Process

Potential participants were identified by a clinician at each site during their regular scheduled appointments at a participating DEP. Those wanting more information met with the site coordinator and were given a brochure on the intervention and a copy of study consent form to review. If interested, the site coordinator would facilitate a phone call between the participant and study research assistant to obtain verbal consent. Participants were then randomized to 1 of 2 arms. Baseline questionnaires were completed over the phone by the research assistant at that time or within 2 weeks of randomization. Patients randomized to the ITG would meet with the site coordinator to receive the phone loaded with the BlueStar app along with a training session designed by the Ontario Telemedicine Network. Participants in the WLC group would arrange an appointment with the site coordinator in 3 months to receive their intervention and training.

### Allocation

Randomization was done in a centralized fashion by the Applied Health Research Centre (AHRC) at the Li Ka Shing Knowledge Institute of St. Michael’s Hospital in Toronto, Canada. Subject randomization was computer generated and stratified by site, using block sizes of 2 or 4, through REDCap [18], a Web-based electronic data entry system at the AHRC. Once the participant completed a baseline questionnaire, the centralized research assistant accessed the randomization sequence and informed the patient of their allocation to receive 1 of 2 treatments with a 1:1 randomization scheme (ITG or WLC).

### Intervention

The intervention was the BlueStar mobile app, designed to act as a virtual coach for patients with T2DM. The app was preloaded onto a cellular network–connected Samsung smartphone (with all other features disabled). The phone was connected to a cellular data plan for internet connectivity and was able to connect to local Wi-Fi networks. If participants used the app without an internet connection, the information was saved and uploaded to the secure server when the phone regained an internet connection. Patients could enter information related to T2DM management into the app, including baseline health, daily blood glucose readings, exercise activity, and food intake (see Multimedia Appendix 1). The app used this information to deliver customized, evidence-based messages in real time that aim to impact motivation, behavior, and education. The messages, based on the Transtheoretical Model of Behavior Change, included educational and affirmational content to encourage sustained behavior changes. Educational messages were aligned with the American Association of Diabetes Educators 7 Standard of Care [19]. The app also facilitated the transfer of data to the user’s clinician through *Smart Visit* reports that provide a clinical overview of current diabetes management including recent blood sugar readings.

Patients in the WLC group received usual diabetes care by the DEP and their primary care physician for the first 3 months of the study. To align with the principles of pragmatic trials, the usual care received was not standardized among participants [20].

### Outcomes and Data Collection

The primary outcome for the trial was glucose control measured by HbA_1c_ levels at 3 months. Secondary outcomes assessed intervention impact on patient self-management, experience of care, and self-reported health utilization using patient-reported outcomes measures and patient-reported experience measures. This included patient self-efficacy measured using 2 validated scales for diabetes, the Problem Areas in Diabetes [21] and the Summary of Diabetes Self-Care Activities [22], as well as quality of life measures using the EuroQol-5D (EQ-5D) [23].

Data were collected centrally by research assistants and inputted into the REDCap database. All outcomes were assessed at 3 and 6 months. Intervention usability, an additional secondary outcome, was evaluated by an adapted version of the Mobile App Rating Scale. App utilization data were routinely collected through the app. Utilization measures include the mean number of engagements per week and the frequency of use of each feature per week.

### Statistical Analysis

Patient characteristics and baseline HbA_1c_ levels were summarized using descriptive statistics, including means and SD for continuous variables and proportions for categorical variables. Data were analyzed according to the intention-to-treat principle. Primary analyses used analysis of covariance (ANCOVA) with all complete cases. A secondary analysis adjusting for study site, length of diabetes diagnosis, ethnicity, and length in DEP was also conducted. A sensitivity analysis to explore the impact of missing data was conducted by identifying all characteristics that significantly differed between those included and not included and then adding these to the primary model with the assumption that the data are at least missing at random. Self-reported health utilization data including hypoglycemic episodes, visits to a primary care physician, visits to a specialist, visits to the emergency department, and hospital admission were converted to binary outcomes (event vs no event) and analyzed using a logistic regression model.

After 6 months, HbA_1c_ levels among those participants in the ITG were compared using a paired *t* test to look for sustained impact of the intervention. App utilization data were analyzed descriptively, including frequency of use (mean uses per week) by site and feature. An exploratory analysis to assess the impact of app usage on 3-month HbA_1c_ and Problem Areas in Diabetes (PAID) scale values was conducted using general linear models that controlled for baseline values.

Power was determined assuming an ANCOVA analysis with an estimate correlation between baseline and follow-up HbA_1c_ measurements of 0.80. The power to detect a difference of 0.7% in HbA_1c_ levels using an SD of 2% between treatment groups at 3 months is 99.7% at a significance level of 5%, based on a sample size of 255 (which assumes a dropout rate of 15% from the target sample size of 300 participants).

## Results

### Study Participants

**Figure 1:**
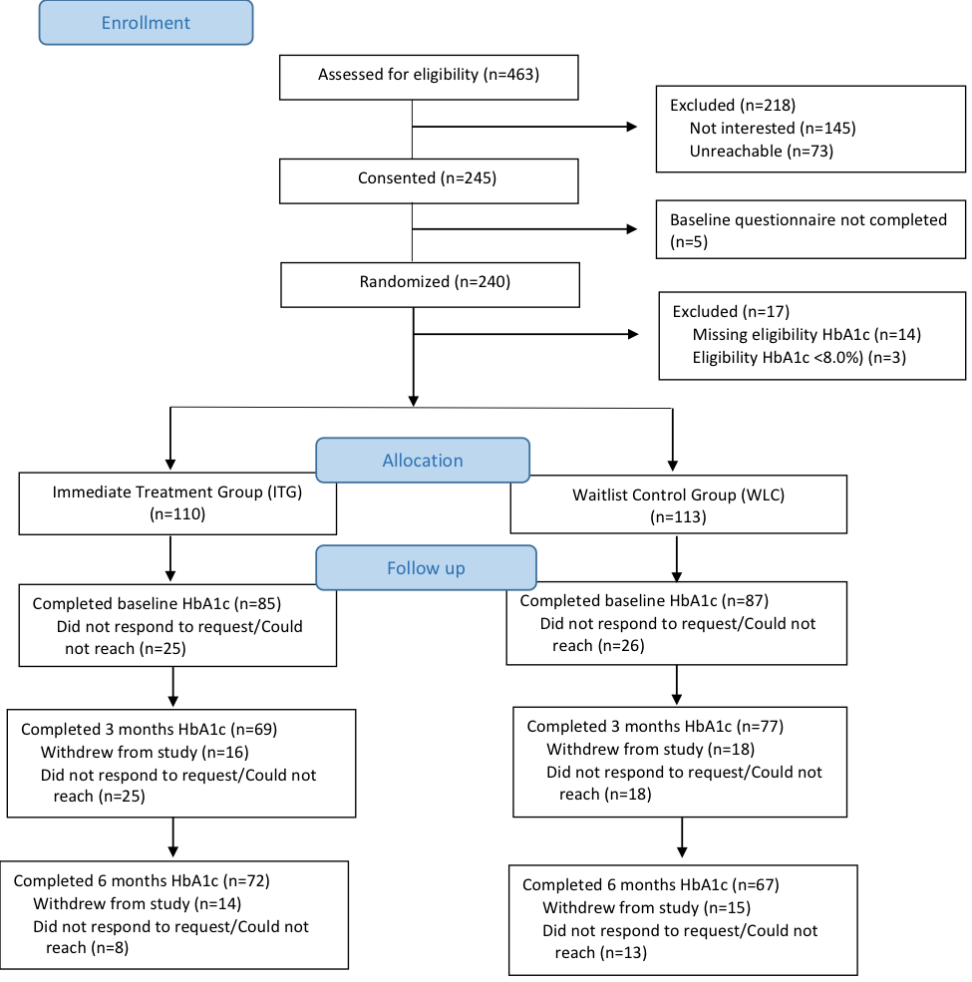
Flowchart of enrollment. HbA_1c_: hemoglobin A_1c_.

Potential participants were identified based on the study criteria and enrolled in the study between June and December 2016. We invited 463 patients; of those, 145 were not interested, 74 were unreachable for follow-up, and 5 did not complete baseline questionnaires (Figure 1). Randomization was completed on 240 participants, but 17 were excluded (8 in the WLC group and 9 in ITG) because of an eligibility HbA_1c_ <8.0%. Thus, 223 participants were included in the study. On follow-up, 77.1% (172/223) of participants completed a baseline HbA_1c_ value, whereas 65.5% (146/223) completed the primary outcome (HbA_1c_ levels at 3 months). A comparison of baseline characteristics shows no significant differences among those who completed the primary outcome versus those who did not, except that nonwhite were less likely to have a 3-month HbA_1c_ value (Multimedia Appendix 2). In total, 120 participants (63 in the WLC group and 57 in the ITG) had both baseline and 3-month HbA_1c_ values completed.

**Table 1:** Baseline characteristics of participants.

Variable		Immediate treatment group (n=110), n (%)	Wait-list control (n=113), n (%)	Overall (N=223), n (%)
Age^a^ (years), mean (SD)		51.5 (10.6)	52.1 (10.7)	51.8 (10.7)
**Sex, n (%)**				
	Male	61 (55.0)	55 (49.0)	116 (52.0)
	Female	48 (44.0)	58 (51.0)	106 (48.0)
	Not specified	1 (0.9)	0 (0.0)	1 (0.0)
**Ethnicity, n (%)**				
	Caucasian	46 (41.8)	50 (44.3)	96 (43.1)
	Non-Caucasian	64 (58.2)	60 (53.0)	124 (55.6)
	Refuse to answer	0 (0.0)	2 (1.8)	2 (0.9)
	Missing	0 (0.0)	1 (0.9)	1 (0.5)
**Education, n (%)**				
	High school or less	32 (29.1)	37 (32.7)	69 (31.0)
	College degree or diploma	49 (46.6)	44 (38.9)	93 (41.7)
	Undergraduate university degree	11 (10.0)	14 (12.4)	25 (11.2)
	Postgraduate degree	5 (4.6)	6 (5.3)	11 (5.0)
	Other	4 (3.6)	8 (7.1)	12 (5.3)
	Not applicable	2 (1.8)	1 (0.9)	3 (1.3)
	Refuse to answer	6 (5.4)	2 (1.8)	8 (3.6)
	Missing	1 (0.9)	1 (0.9)	2 (0.9)
**Household income (Can $), n (%)**				
	<$35,000	30 (27.3)	24 (21.2)	54 (24.3)
	$35,000-$50,000	10 (9.1)	24 (21.2)	34 (15.1)
	>$50,000-$80,000	23 (20.9)	17 (15.0)	40 (18.0)
	>$80,000-$150,000	17 (15.5)	21 (18.6)	38 (17.0)
	>$150,000	6 (5.5)	5 (4.4)	11 (5.0)
	Not applicable	9 (8.2)	4 (3.5)	13 (5.8)
	Refuse to answer	15 (13.6)	16 (14.2)	31 (13.9)
	Missing	0 (0.0)	2 (1.8)	2 (0.9)
**Time since diabetes diagnosis, n (%)**				
	0-6 months	16 (14.6)	24 (21.2)	40 (18)
	>6 months to 2 years	25 (22.7)	27 (23.9)	52 (23)
	>2-5 years	26 (23.6)	13 (11.5)	39 (18)
	5+ years	41 (37.3)	47 (41.6)	88 (40)
	Unsure	1 (0.9)	2 (1.8)	3 (1)
	Missing	1 (0.9)	0 (0.0)	1 (0.0)
Baseline value for HbA_1c_^b^, mean (SD)		8.89 (1.82)	9.03 (1.53)	8.96 (1.68)
**Time in diabetes education, n (%)**				
	New patient	35 (31.8)	41 (363)	76 (34.1)
	1-6 months	15 (13.6)	22 (19.5)	37 (16.6)
	>6-12 months	22 (20.0)	19 (16.8)	41 (18.4)
	1+ years	36 (32.7)	31 (27.4)	67 (30.1)
	Unsure	1 (0.9)	0 (0)	1 (0.4)
	Missing	1 (0.9)	0 (0)	1 (0.4)
**Insulin use, n (%)**				
	Yes	50 (45.0)	60 (53.0)	110 (49.0)
	No	60 (55.0)	53 (47.0)	113 (51.0)

^a^N=222.

^b^HbA_1c_: hemoglobin A_1c_, N=172.

Table 1 summarizes the demographic characteristics of the study population. There were no significant differences in patient characteristics including age, gender, ethnicity, education, and household income. About 18.0% (40/223) of participants were diagnosed with T2DM within the last 6 months, whereas 39.5% (88/223) had a diagnosis of T2DM for over 5 years. The average HbA_1c_ level for the study population was 8.96% (SD 1.68) and was similar between the 2 study arms, and the use of insulin was similar between the 2 groups. Additional clinical features, including baseline medication usage and comorbidities, were similar across study arms (Multimedia Appendix 3).

### Outcomes

#### Primary Outcome

**Figure 2:**
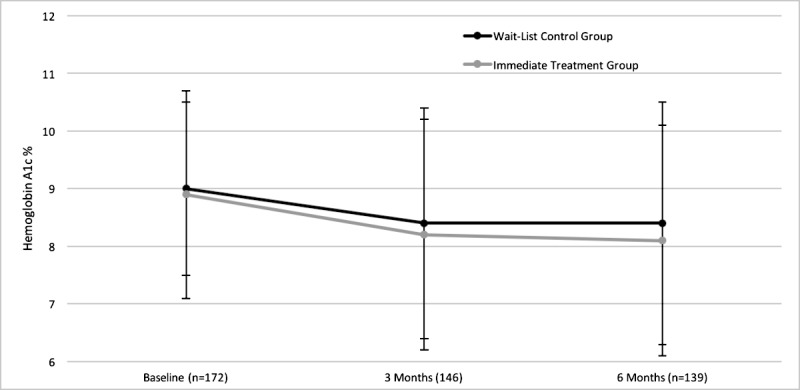
Mean HbA_1c_ (hemoglobin A_1c_) values for intervention and control groups from baseline to 6 months.

Figure 2 shows the HbA_1c_ levels for patients in the ITG and WLC group at baseline, 3 months, and 6 months. At 3 months, the unadjusted mean HbA_1c_ values were 8.22% for the ITG and 8.41% for the WLC group. The results of an ANCOVA controlling for baseline values of 120 participants (63 WLC and 57 ITG) did not show evidence of impact on HbA_1c_ levels at 3 months for those in the ITG (mean difference [ITG-WLC] −0.42, 95% CI −1.05 to 0.21; *P*=.19). This nonsignificant difference between groups persisted after adjustment for study site, length of diabetes diagnosis, ethnicity, and length of time spent in the DEP (mean difference [ITG−WLC] −0.12, 95% CI −0.71 to 0.47).

Baseline characteristics were compared between the 120 participants included in the above model with the 103 participants who had incomplete HbA_1c_ data and were excluded to determine whether the 2 subgroups differed systematically from one another. After adjusting the main ANCOVA model for all covariates found to be associated with complete versus incomplete HbA_1c_ data (ie, site, time since diabetes diagnosis, ethnicity, antidepressant use, dyslipidemia, and obesity), the effect of treatment on 3-month HbA_1c_ levels remained statistically insignificant (least squares adjusted mean difference −0.33, 95% CI −0.99 to 0.34).

An exploratory analysis of ITG participants investigating the impact of app usage on 3-month HbA_1c_ levels while adjusting for baseline HbA_1c_ levels was conducted using an ANCOVA. Only 57 participants were complete cases and included in the regression. Each additional day of app use corresponded with a 0.016-point decrease in participant’s 3-month HbA_1c_ levels (95% CI −0.03 to −0.003; *P*=.02). In other words, 25 days of additional use of the app corresponded with an HbA_1c_ reduction of 0.4%. A correlation matrix of this analysis (Multimedia Appendix 4) found a weak correlation between increased use of the exercise feature with lower HbA_1c_ levels at 3 months (ρ_s_=−0.33; *P*=.01). An analysis of ITG participants, using a paired *t* test, did not show a statistically significant difference in HbA_1c_ levels between 3 and 6 months (mean difference 0.16, 95% CI −0.48 to 0.81).

#### Secondary Outcomes

Overall, there was no difference in patient-reported diabetes self-care behaviors (measured by PAID and Summary of Diabetes Self-Care Activities-6) or general health status (measured by EQ-5D) at 3 months between intervention arms in both the unadjusted and adjusted models (Multimedia Appendix 5). Furthermore, there was no difference in health care utilization at 3 months between groups (Table 2). An exploratory analysis of 63 ITG participants investigating the impact of app usage on PAID score levels at 3 months, adjusting for baseline scores, did not show evidence of significance (95% CI −0.28 to 0.091; *P*=.32).

**Table 2:** Health service utilization.

Outcome (N=baseline/3 month)	ITG^a^ (% with event)		WLC^b^ (% with event)		Odds ratio (95% CI)	*P* value
	Baseline, n (%)	3 months, n (%)	Baseline, n (%)	3 months, n (%)		
Emergency deparment visits (223/139)	21 (19.0)	5 (7.5)	12 (10.6)	5 (6.8)	1.11 (0.03-0.18)	0.86
Hypoglycemic episodes (223/139)	32 (29.0)	21 (31.8)	25 (22.1)	15 (20.5)	1.80 (0.84-3.89)	0.13
Hospital admission (223/139)	23 (20.9)	9 (13.6)	16 (14.1)	4 (5.4)	2.72 (0.78-9.91)	0.11
Visit to primary care provider (222/138)	95 (86.3)	57 (86.3)	103 (91.1)	64 (87.6)	0.79 (0.29-2.19)	0.65
Visit to specialist (223/139)	78 (70.9)	37 (56.0)	70 (61.9)	46 (63.0)	0.75 (0.38-1.48)	0.40

^a^ITG: immediate treatment group.

^b^WLC: wait-list control.

### Mobile App Utilization and Satisfaction

**Figure 3:**
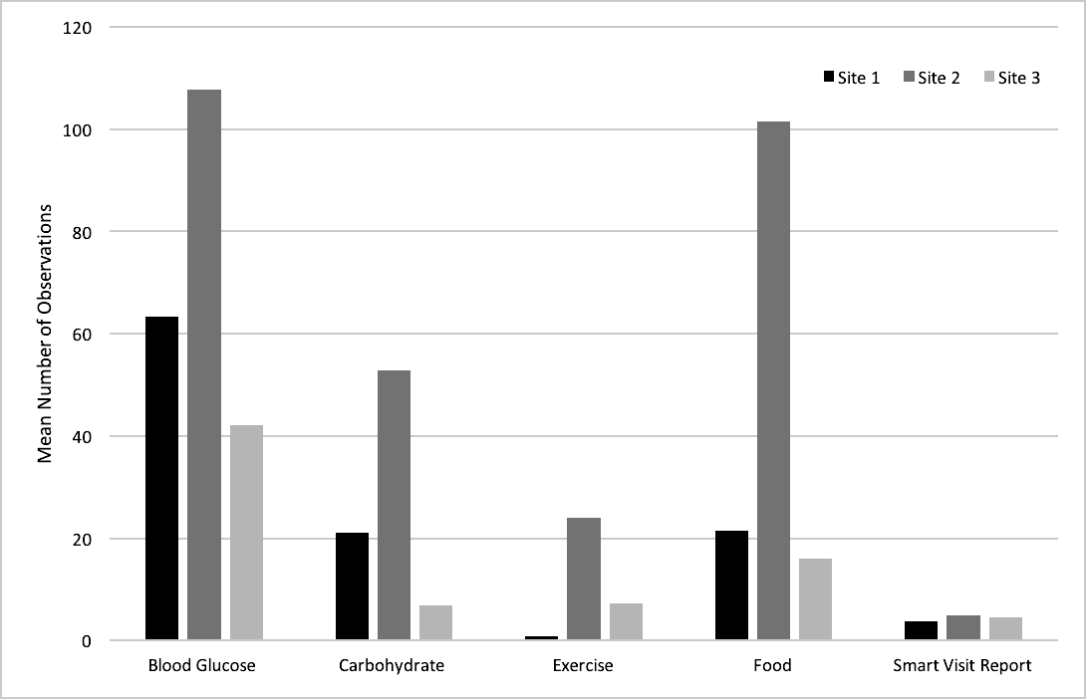
Mean number of observations recorded by feature and site for immediate treatment group (ITG) participants over 14 weeks.

Overall, there was low app utilization among ITG participants with a mean number of log-in days of 42.4 (SD 52.1) over 26 weeks, of which 46.4% (51/110) of participants used the app for 10 days or less. There was a small percentage of high users, with 18.2% (20/110) of participants using the app 100 days or more over a 182-day period. Multimedia Appendix 6 shows average number of log-in days among ITG participants over 26 weeks, showing significant decreasing mean usage over time. Blood glucose tracking was the most utilized feature with an average of 76.6 entries over 14 weeks (SD 96.59), whereas exercise tracking was the least utilized (mean 26.7 [SD 53.4]; see Figure 3). Of note, this graph also shows high variability in usage by site. Over the first 14 weeks, site 2 showed the highest number of log-in days by participants (median 36; interquartile range [IQR] 10.5-124), whereas participants from site 3 used the app significantly less (median 9; IQR 6-51). Site 1 has intermediate usage (median 17; IQR 7-72). Users with a diagnosis of diabetes in the last 6 months were the most engaged as assessed by days of log-in (median 24.5; IQR 8.7-73.5), whereas those with a diagnosis for over 5 years also had high engagement (median 18; IQR 8-86).

User ratings were completed by 105 participants to assess satisfaction with the app. Almost half of those who responded (45.7, 48/105) stated they would recommend the app to all people like them. Moreover, 41.0% (43/105) stated they would use the app 50 times or more if they continued to have access to it. About half (53.3%, 56/105) gave the app a rating of 4 to 5 stars of 5, whereas 39.0% (41/105) gave the app a rating of 3 stars. When asked if they would be willing to pay for the app, the majority of participants (55.2%, 58/105) stated they would not.

## Discussion

### Principal Findings

The aim of this study was to evaluate the clinical impact of the BlueStar app for diabetes self-management in a real-world multisite implementation. The results showed no difference between intervention and control arms for the primary clinical outcome of glycemic control as measured by HbA_1c_. Furthermore, we found no intervention effect on secondary outcomes measuring diabetes self-efficacy, quality of life, and health care utilization behaviors. Of note, there was relative low use of the app overall, with almost half of intervention group users having minimal engagement with the app. Many app features were poorly utilized, including diet and exercise tracking, which have previously shown to play an important role in T2DM self-management [10]. There was a small number of highly engaged users, and exploratory analysis suggests a correlation with app usage, and improvement in HbA_1c_ levels at 3 months analysis suggests that 25 days of usage associated with an improvement in HbA_1c_ level by 0.4%, a clinically significant change [24].

To our knowledge, this is the largest pragmatic multisite trial of evaluation of a mobile app for self-management of T2DM, and the results are in contrast to prior published studies of mobile app for diabetes self-management. These studies of mobile apps for T2DM largely consist of small single-site studies with a homogeneous population [8,25]. A meta-analysis of 10 studies of T2DM apps reported a medium reduction in HbA_1c_ level of 0.55% among those using an app, with all studies reporting some positive benefit. However, these tended to have small study populations, and 8 of the 10 studies included additional ongoing feedback from the PCP as part of the intervention [8]. Similarly, a previous study of the same mobile app, which showed significant decrease in HbA_1c_ level among intervention participants, was conducted with only 30 participants. Moreover, in that study, the intervention arm received the mobile app plus multiple follow-up interactions from the research team to the physician and patient [13]. This large multisite study likely represents a more realistic assessment of impact for a diabetes health app across a health system than smaller, higher touch, single-site studies.

Our findings suggest that when evaluating a mobile app for chronic disease management, it is important to ask not only if the app works but also in what context, for which patients, and how to promote ongoing engagement of use. Overall, there was low usage of the app among participants. However, results indicate that contextual factors, particularly site, had a significant impact on overall usage of the app. App usage overall and across features was almost twice as high among site 2 compared with site 3. Despite comprehensive implementation protocols, there were substantial differences in time spent training clinicians, time training patients, and ongoing engagement with patients between clinical test sites, with the highest use site spending the greatest time and resources on implementation. In addition, it is increasingly evident that digital health apps designed to improve chronic disease self-management require ongoing patient engagement as a key determinant of clinical impact [26-30]. Therefore, a successful implementation and evaluation of these apps require careful consideration of factors that impact patient app utilization [30]. In this study, patients with a new diagnosis of T2DM had significantly higher usage than those who were diagnosed more than 6 months prior. Previous studies have shown patient factors including age, internal motivation, and personal values impact utilization of mobile health technologies [31]. This aligns with the results of a qualitative evaluation conducted with a subset of patients from this study. It found that perceived self-efficacy, competing priorities, and beliefs about the usefulness of virtual solutions had a significant impact on app utilization [17].

A recent systematic review of factors that impact engagement with digital health interventions highlighted the importance of both patient factors and engagement and recruitment methods [32]. Several recent studies of apps for T2DM have emphasized the importance of an implementation that includes a strong clinical endorsement and ongoing clinical support to increase overall usage [33,34]. A qualitative study of patients who dropped out of a study evaluating a self-management app for T2DM cited lack of clinician support as the primary reason for leaving. Our complementary qualitative study found participants with high app utilization identified the health care provider and/or site coordinator as a significant source of support in app adoption. These align with our quantitative findings that variation in app usage across sites was at least in part driven by variation in implementation. Future implementations of digital health apps would benefit from a clear effort to include factors that improve engagement, including a strong clinical endorsement, ongoing physician involvement, and patient reminders [35].

### Limitations

Several limitations to this study warrant discussion. Importantly, the study was underpowered to detect small but potentially still important differences in HbA_1c_ levels. The studies’ high dropout rate of 34.5% (77/223), while in line with prior electronic health (eHealth) studies, may have led to an underestimation of the clinical impact among participants [36,37]. There were several study design factors that likely contributed to the low app usage and lack of a detected intervention effect. Instead of downloading the app, participants were given the intervention on a second phone they used for the duration of the study in an attempt to standardize implementation by the funder. However, the use of a second phone to deliver eHealth interventions has been a noted barrier to usage in previous studies, and future mobile app evaluations would likely benefit from allowing participants to use their own smartphones when possible [28]. Given previous evidence on the benefits of strong primary care participation in diabetes self-management apps, the use of DEPs as the primary site of recruitment likely had a negative impact on enrollment, usage, and clinical impact [38,39]. Clinicians at the selected DEPs did not have regular communication with PCPs, and therefore, there was no robust pathway to report use of the app or possible treatment enhancements to the PCP. Future implementations of this, or similar apps, would likely benefit from strong primary care involvement throughout the study who can support self-management through direct treatment changes including medication titration. Finally, as discussed previously, significant variations in implementation across sites likely also had significant impact on site usage and overall ability to detect a clinical effect.

### Conclusions

In this large real-world evaluation of a mobile app for diabetes self-management, we found no significant difference in HbA_1c_ levels between the intervention and control groups. Future research into the patient and site-specific factors that would increase app utilization would be warranted.

## Appendix

Multimedia Appendix 1 BlueStar mobile app interface.

Multimedia Appendix 2 Comparison of baseline characteristics among those with and without an HbA_1c_ (hemoglobin A_1c_) value at 3 months.

Multimedia Appendix 3 Baseline clinical characteristics of participants.

Multimedia Appendix 4 Spearman correlation coefficients of immediate treatment group (ITG) participants with complete cases (n=57).

Multimedia Appendix 5 Patient-reported outcome and experience measures.

Multimedia Appendix 6 Mean app usage among immediate treatment group (ITG) users over time.

Multimedia Appendix 7 CONSORT‐EHEALTH checklist (V.1.6.1).

## Abbreviations

AHRC: Applied Health Research Centre
ANCOVA: analysis of covariance
DEP: diabetes education program
eHealth: electronic health
EQ-5D: EuroQol-5D
HbA_1c_: hemoglobin A_1c_
IQR: interquartile range
ITG: immediate treatment group
OHIP: Ontario Health Insurance Program
PAID: Problem Areas in Diabetes
PCP: primary care provider
T2DM: type 2 diabetes mellitus
WLC: wait-list control
