# Supplementary material for: Mobile App for Improved Self-Management of Type 2 Diabetes: Multicenter Pragmatic Randomized Controlled Trial
Source: JMIR Mhealth Uhealth. 2019 Jan 10;7(1):e10321. doi: 10.2196/10321 (PMC6329896; doi:10.2196/10321)
Supplement: Multimedia Appendix 1 [file mhealth_v7i1e10321_app1.pdf]

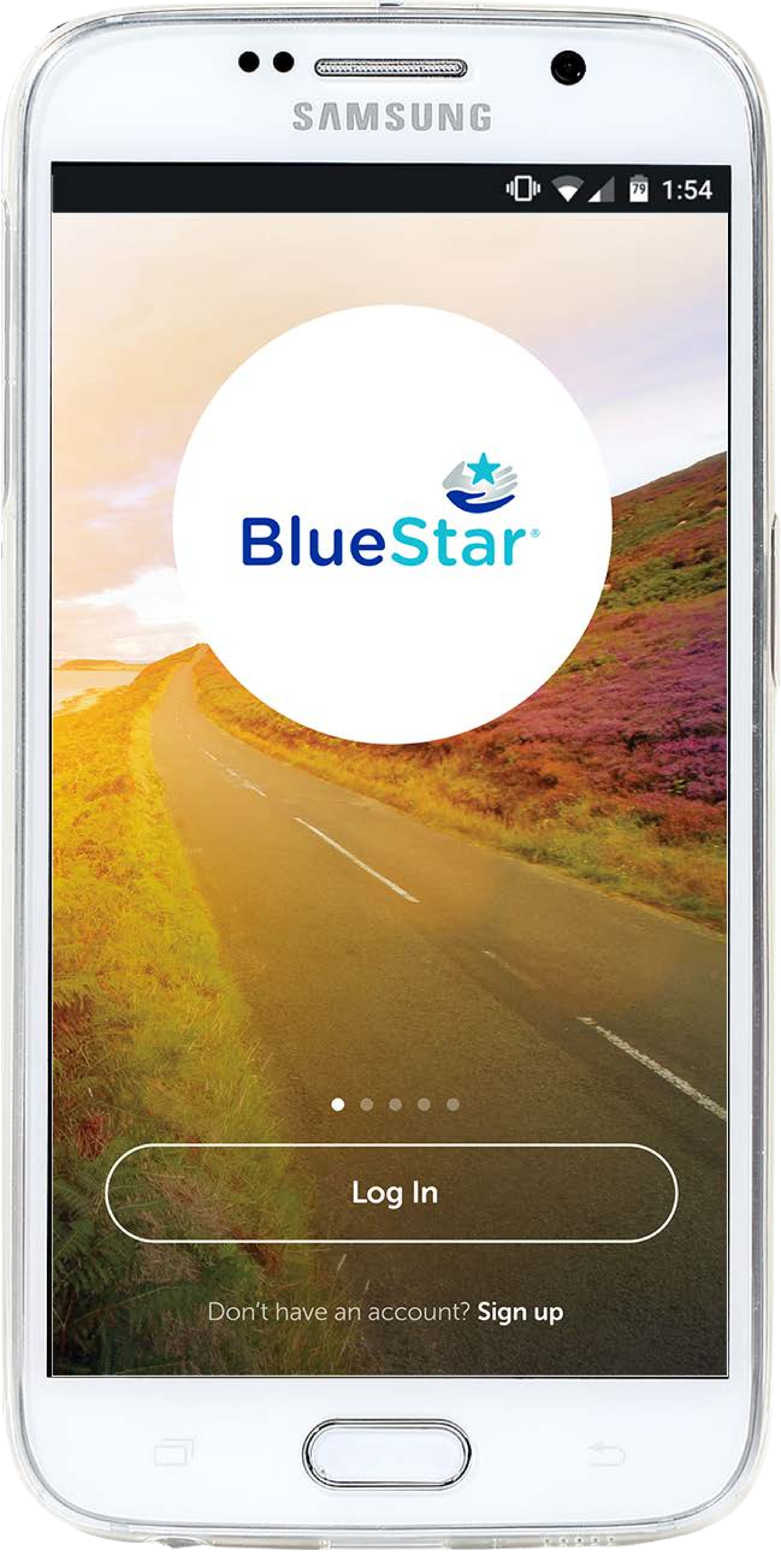
SAMSUNG
1:54
BlueStar
Log In
Don't have an account? Sign up

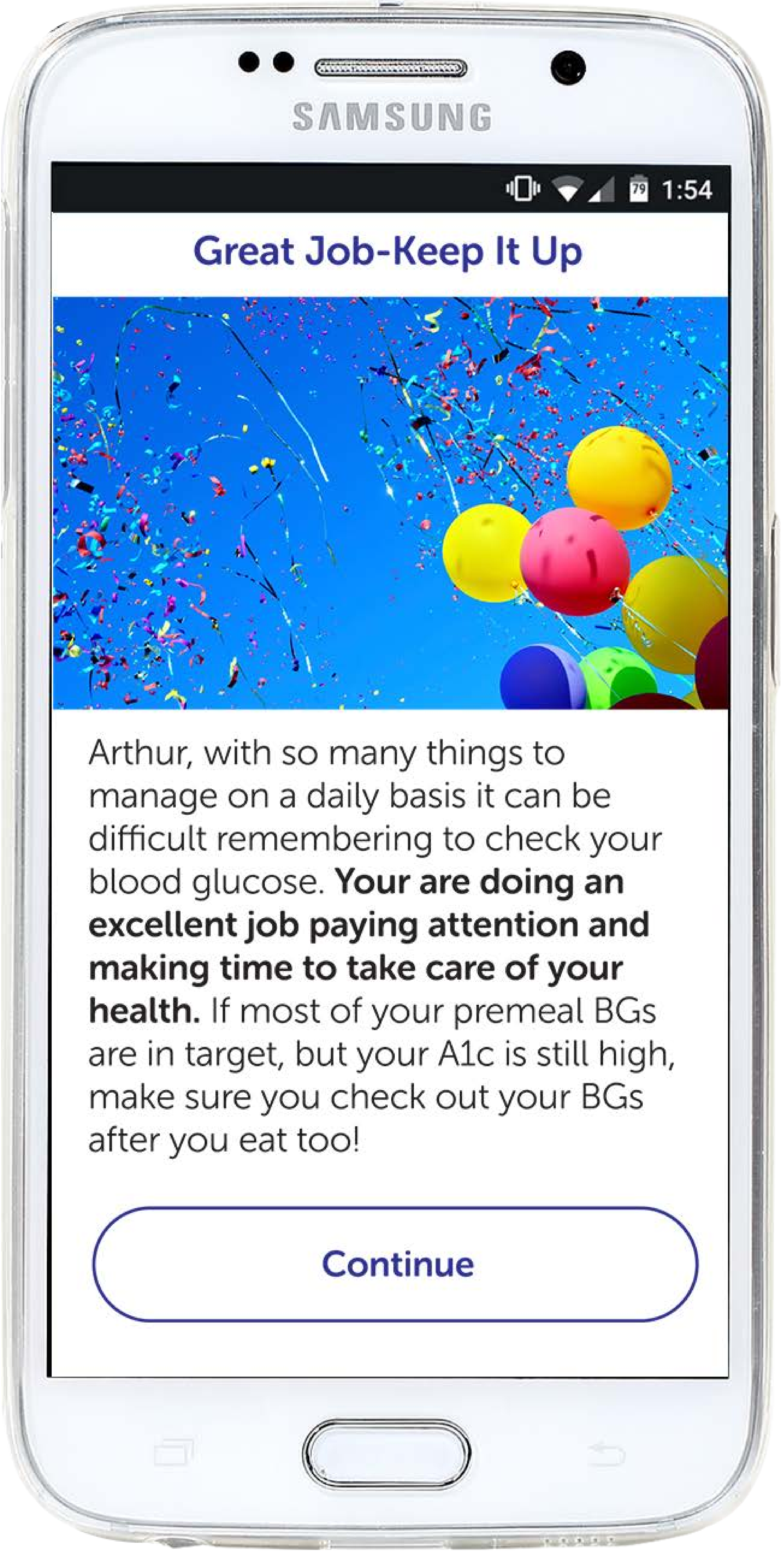
SAMSUNG
1:54
Great Job-Keep It Up
Arthur, with so many things to manage on a daily basis it can be difficult remembering to check your blood glucose. **Your are doing an excellent job paying attention and making time to take care of your health.** If most of your premeal BGs are in target, but your A1c is still high, make sure you check out your BGs after you eat too!
Continue

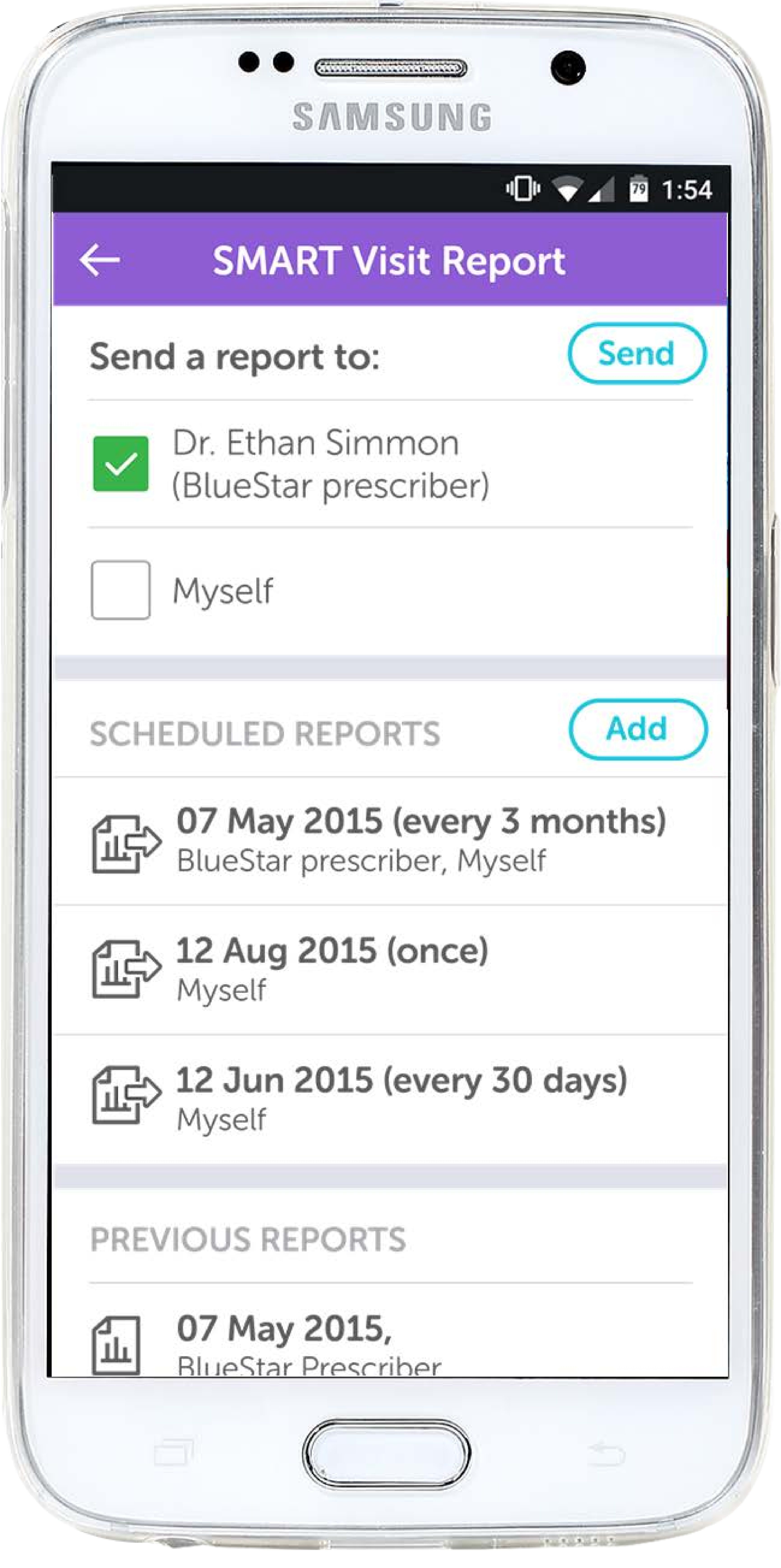
SAMSUNG
1:54
SMART Visit Report
Send a report to:
Send
Dr. Ethan Simmon
(BlueStar prescriber)
Myself
SCHEDULED REPORTS
Add
07 May 2015 (every 3 months)
BlueStar prescriber, Myself
12 Aug 2015 (once)
Myself
12 Jun 2015 (every 30 days)
Myself
PREVIOUS REPORTS
07 May 2015,
BlueStar Prescriber

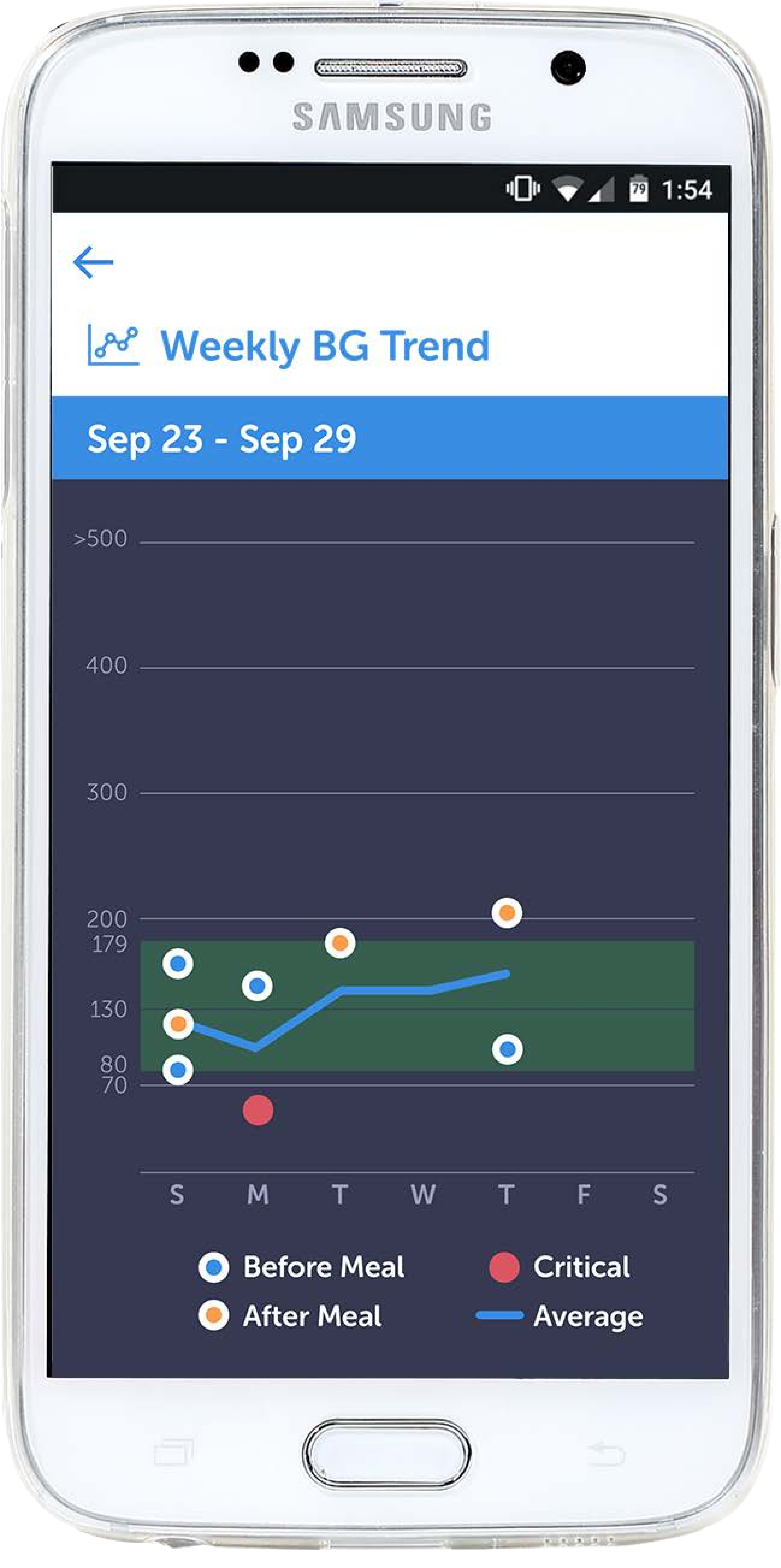
SAMSUNG
1:54
Weekly BG Trend
Sep 23 - Sep 29
>500
400
300
200
179
130
80
70
S
M
T
W
T
F
S
Before Meal
After Meal
Critical
Average
