# Supplementary material for: Mobile App for Improved Self-Management of Type 2 Diabetes: Multicenter Pragmatic Randomized Controlled Trial
Source: JMIR Mhealth Uhealth. 2019 Jan 10;7(1):e10321. doi: 10.2196/10321 (PMC6329896; doi:10.2196/10321)
Supplement: Multimedia Appendix 2 [file mhealth_v7i1e10321_app2.pdf]

Variable	No HbA1c N=77	Had HbA1c N=146	Combined N=223
**Age (N=222),** Mean ±SD	51.3±11.5	52.1±10.2	51.8±10.7
**Sex (N=223)**			
Male	45 (58%)	71 (49%)	116 (52%)
Female	32 (42%)	74 (51%)	106 (48%)
Transgendered	0 (0%)	0 (0%)	0 (0%)
Not specified	0 (0%)	1 (1%)	1 (0%)
**Ethnicity (N=222)**			
Caucasian	22 (29%)	74 (51%)	96 (43%)
Non-Caucasian	55 (71%)	69 (48%)	124 (56%)
Refuse to answer	0 (0%)	2 (1%)	2 (1%)
**Education (N=221)**			
High school or less	22 (29%)	47 (33%)	69 (31%)
College degree/diploma	36 (47%)	57 (40%)	93 (42%)
Undergraduate university degree	5 (6%)	20 (14%)	25 (11%)
Post Graduate degree	2 (3%)	9 (6%)	11 (5%)
Other	5 (6%)	7 (5%)	12 (5%)
Not applicable	1 (1%)	2 (1%)	3 (1%)
Refuse to answer	6 (8%)	2 (1%)	8 (4%)
**Household income (N=221)**			
Under $35,000	16 (21%)	38 (26%)	54 (24%)
$35,000-$50,000	10 (13%)	24 (17%)	34 (15%)
>$50,000-$80,000	12 (16%)	28 (19%)	40 (18%)
>$80,000-$150,000	15 (20%)	23 (16%)	38 (17%)
>$150,000	4 (5%)	7 (5%)	11 (5%)
Not applicable	9 (12%)	4 (3%)	13 (6%)
Refuse to answer	10 (13%)	21 (14%)	31 (14%)
**Time since diabetes diagnosis (N=222)**			
0-6 months	11 (14%)	29 (20%)	40 (18%)
>6 months – 2 years	23 (30%)	29 (20%)	52 (23%)
>2-5 years	19 (25%)	20 (14%)	39 (18%)
5+ years	22 (29%)	66 (45%)	88 (40%)
Unsure	1 (1%)	2 (1%)	3 (1%)
**Time in diabetes education (N=222)**			
New Patient	27 (36%)	49 (34%)	76 (34%)
1-6 months	12 (16%)	25 (17%)	37 (17%)
>6-12months	12 (16%)	29 (20%)	41 (18%)
1+ years	24 (32%)	43 (29%)	67 (30%)
Unsure	1 (1%)	0 (0%)	1 (0%)
**Insulin use (N=223)**			
Yes	40 (52%)	70 (48%)	110 (49%)
No	37 (48%)	76 (52%)	113 (51%)

Appendix 2: Comparison of Baseline Characteristics among those with and without a HbA1c value at 3 months
