# Supplementary material for: Mobile App for Improved Self-Management of Type 2 Diabetes: Multicenter Pragmatic Randomized Controlled Trial
Source: JMIR Mhealth Uhealth. 2019 Jan 10;7(1):e10321. doi: 10.2196/10321 (PMC6329896; doi:10.2196/10321)
Supplement: Multimedia Appendix 3 [file mhealth_v7i1e10321_app3.pdf]

Variable	ITG (N=110)	WLC (N=113)	Overall (N=223)
**Oral anti-diabetic agent use**			
Yes	103 (91.0%)	97 (88.0%)	200 (90.0%)
No	10 (9.0%)	13 (12%)	23 (10%)
**Analogues injection use**			
Yes	5 (4.0%)	3 (3.0%)	8 (4.0%)
No	108 (96.0%)	107 (97.0%)	215 (96.0%)
**Anti-hypertensive use**			
Yes	37 (33.0%)	35 (32.0%)	72 (32.0%)
No	76 (67.0%)	75 (68.0%)	151 (68.0%)
**Anti-depressant use**			
Yes	9 (8.0%)	13 (12.0%)	22 (10.0%)
No	104 (92.0%)	97 (88.0%)	201 (90.0%)
**Statin use**			
Yes	43 (38.0%)	31 (28.0%)	74 (33.0%)
No	70 (62.0%)	79 (72.0%)	149 (67.0%)
**CVD**			
Yes	19 (17.0%)	20 (18.0%)	39 (17.0%)
No	94 (83.0%)	90 (82.0%)	184 (83.0%)
**CKD**			
Yes	5 (4.0%)	3 (3.0%)	8 (4.0%)
No	108 (96.0%)	107 (97.0%)	215 (96.0%)
**Hypertension**			
Yes	47 (42.0%)	50 (45.0%)	97 (43.0%)
No	66 (58.0%)	60 (55.0%)	126 (57.0%)
**Dyslipidemia**			
Yes	58 (51.0%)	49 (45.0%)	107 (48.0%)
No	55 (49.0%)	61 (55.0%)	116 (52.0%)
**Obesity**			
Yes	35 (31.0%)	37 (34.0%)	72 (32.0%)
No	78 (69.0%)	73 (66.0%)	151 (68.0%)
**Depression or anxiety**			
Yes	13 (11.5%)	18 (16.0%)	31 (13.8%)
No	100 (88.5%)	92 (84.0%)	192 (86.2%)

Appendix 3: Baseline Clinical Characteristics of Participants
