# Supplementary material for: Mobile App for Improved Self-Management of Type 2 Diabetes: Multicenter Pragmatic Randomized Controlled Trial
Source: JMIR Mhealth Uhealth. 2019 Jan 10;7(1):e10321. doi: 10.2196/10321 (PMC6329896; doi:10.2196/10321)
Supplement: Multimedia Appendix 4 [file mhealth_v7i1e10321_app4.pdf]

Appendix 4: Spearman Correlation Coefficients of ITG participants with complete cases (n=57)

Spearman Correlation Coefficients (N = 57)								
Prob > |r| under H0: Rho=0								
			Number of uses of the feature on the app					
	Baseline	3 months	Total days with at least one app use	Carbs	Food Tracking	Exercise	Blood Glucose Tracking	Smart visit Reports
Baseline	1.00000	0.43786 (0.0007)	-0.25201 (0.0586)	-0.18759 (0.1623)	-0.21553 (0.1074)	-0.32921 (0.0124)	-0.19348 (0.1493)	-0.22724 (0.0891)
3 months	0.43786 (0.0007)	1.00000	-0.04191 (0.7569)	0.14868 (0.2697)	0.08632 (0.5232)	0.05389 (0.6906)	0.02637 (0.8456)	-0.19322 (0.1498)
Total days with at least one app use	-0.04191 (0.7569)	-0.25201 (0.0586)	1.00000	0.44023 (0.0006)	0.35446 (0.0068)	0.41117 (0.0015)	0.83291 (<.0001)	0.35345 (0.0070)
Carbs	0.14868 (0.2697)	-0.18759 (0.1623)	0.44023 (0.0006)	1.00000	0.90456 (<.0001)	0.58421 (<.0001)	0.45985 (0.0003)	0.33682 (0.0104)
Food Tracking	0.08632 (0.5232)	-0.21553 (0.1074)	0.35446 (0.0068)	0.90456 (<.0001)	1.00000	0.61986 (<.0001)	0.36811 (0.0048)	0.30241 (0.0222)
Exercise	0.05389 (0.6906)	-0.32921 (0.0124)	0.41117 (0.0015)	0.58421 (<.0001)	0.61986 (<.0001)	1.00000	0.43547 (0.0007)	0.38100 (0.0035)
Blood Glucose Tracking	0.02637 (0.8456)	-0.19348 (0.1493)	.83291 (<.0001)	0.45985 (0.0003)	0.36811 (0.0048)	0.43547 (0.0007)	1.00000	0.26059 (0.0503)
Smart visit Reports	-0.19322 (0.1498)	-0.22724 (0.0891)	0.35345 (0.0070)	0.33682 (0.0104)	0.30241 (0.0222)	0.38100 (0.0035)	0.26059 (0.0503)	1.00000
