# Supplementary material for: Mobile App for Improved Self-Management of Type 2 Diabetes: Multicenter Pragmatic Randomized Controlled Trial
Source: JMIR Mhealth Uhealth. 2019 Jan 10;7(1):e10321. doi: 10.2196/10321 (PMC6329896; doi:10.2196/10321)
Supplement: Multimedia Appendix 5 [file mhealth_v7i1e10321_app5.pdf]

Appendix 5: Patient Reported Outcome and Experience Measures

Outcome	ITG		WLC		Unadjusted Model Coefficient (95% CI)	Adjusted Model Coefficient (95% CI)
	Baseline (Mean±SD)	3 months (Mean±SD)	Baseline (Mean±SD)	3 months (Mean±SD)		
PAID	29.6 ±22.9	30.7 ± 24.7	25.9 ± 20.1	29.9 ± 20.7	-1.84 (-8.75, 5.07)	-4.73 (-11.88, 2.42)
EQ5D VAS	64.6 ± 21.6	70.5 ± 20.8	67.2 ± 16.8	68.3 ± 18.6	3.02 (-2.79, 8.83)	2.39 (-3.21, 7.99)
Eq5D Index	0.77 ± 0.19	0.77 ± 0.18	0.81 ± 0.15	0.81 ± 0.14	-0.02 (-0.06, 0.03)	-0.010 (-0.05, 0.029)
SDSCA – General Diet	4.16 ± 2.04	4.24 ± 1.91	4.17 ± 2.19	4.05 ± 2.29	0.14 (-0.47, 0.75)	0.19 (-0.47, 0.85)
SDSCA – Specific Diet	4.38 ± 1.69	4.24 ± 1.51	4.54 ± 1.51	4.41 ± 1.68	-0.15 (-0.60, 0.31)	-0.051 (-0.54, 0.44)
SDSCA – Exercise	2.93 ± 2.33	3.15 ± 2.31	2.75 ± 2.20	2.86 ± 2.31	0.031 (-0.68, 0.74)	0.19 (-0.57, 0.95)
SDSCA – Blood Glucose	4.69 ± 2.53	3.99 ± 2.51	4.88 ± 2.40	4.18 ± 2.55	-0.29 (-1.02, 0.43)	-0.19 (-0.94, 0.56)
SDSCA – Foot Care	2.52 ± 2.67	2.46 ± 2.47	3.26 ± 2.47	3.16 ± 2.58	-0.098 (-0.80, 0.61)	-0.0049 (-0.76, 0.75)
SDSCA - # of Cigarettes	2.49 ± 7.46	1.61 ± 5.13	1.22 ± 4.83	1.27 ± 4.77	1.30 (0.44, 3.80)*	not possible due to low event number

*Odds Ratio
