# Supplementary figures and images for: Mobile App for Improved Self-Management of Type 2 Diabetes: Multicenter Pragmatic Randomized Controlled Trial
Source: JMIR Mhealth Uhealth. 2019 Jan 10;7(1):e10321. doi: 10.2196/10321 (PMC6329896; doi:10.2196/10321)

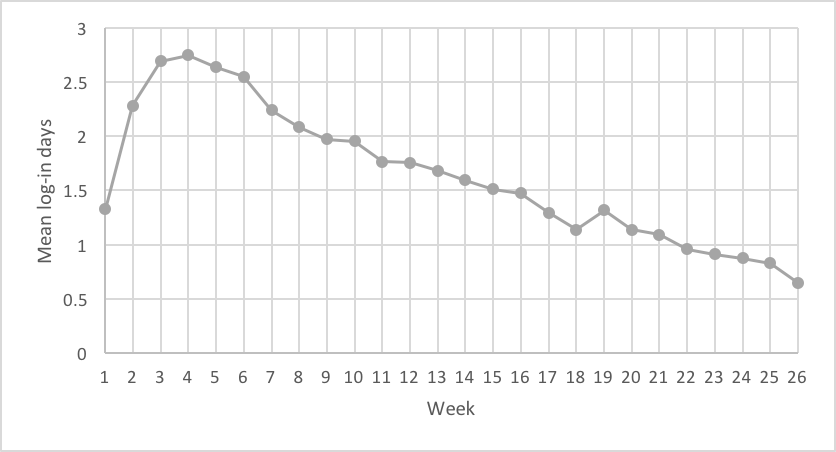

Supplement: Multimedia Appendix 6 [file mhealth_v7i1e10321_app6.png]
